# Genotype-Specific Lesion Growth Rates in Stargardt Disease

**DOI:** 10.3390/genes12121981

**Published:** 2021-12-14

**Authors:** Rachael C. Heath Jeffery, Jennifer A. Thompson, Johnny Lo, Tina M. Lamey, Terri L. McLaren, Ian L. McAllister, Ian J. Constable, John N. De Roach, Fred K. Chen

**Affiliations:** 1 Centre for Ophthalmology and Visual Science (Incorporating Lions Eye Institute), The University of Western Australia, Nedlands, WA 6009, Australia; rachaelheathjeffery@lei.org.au (R.C.H.J.); tina.lamey@health.wa.gov.au (T.M.L.); terri.mclaren@health.wa.gov.au (T.L.M.); ianmcallister@lei.org.au (I.L.M.); ian.constable@lei.org.au (I.J.C.); john.deroach@health.wa.gov.au (J.N.D.R.); 2 Department of Ophthalmology, Royal Perth Hospital, Perth, WA 6000, Australia; 3 Australian Inherited Retinal Disease Registry and DNA Bank, Department of Medical Technology and Physics, Sir Charles Gairdner Hospital, Nedlands, WA 6009, Australia; jennifer.thompson3@health.wa.gov.au; 4 School of Science, Edith Cowan University, Joondalup, WA 6027, Australia; j.lo@ecu.edu.au

**Keywords:** Stargardt disease, *ABCA4*, fundus autofluorescence, growth rate, natural history study

## Abstract

Reported growth rates (GR) of atrophic lesions in Stargardt disease (STGD1) vary widely. In the present study, we report the longitudinal natural history of patients with confirmed biallelic *ABCA4* mutations from five genotype groups: c.6079C>T, c.[2588G>C;5603A>T], c.3113C>T, c.5882G>A and c.5603A>T. Fundus autofluorescence (AF) 30° × 30° images were manually segmented for boundaries of definitely decreased autofluorescence (DDAF). The primary outcome was the effective radius GR across five genotype groups. The age of DDAF formation in each eye was calculated using the x-intercept of the DDAF effective radius against age. Discordance between age at DDAF formation and symptom onset was compared. A total of 75 eyes from 39 STGD1 patients (17 male [44%]; mean ± SD age 45 ± 19 years; range 21–86) were recruited. Patients with c.3113C>T or c.6079C>T had a significantly faster effective radius GR at 0.17 mm/year (95% CI 0.12 to 0.22; *p* < 0.001 and 0.14 to 0.21; *p* < 0.001) respectively, as compared to those patients harbouring c.5882G>A at 0.06 mm/year (95% CI 0.03–0.09), respectively. Future clinical trial design should consider the effect of genotype on the effective radius GR and the timing of DDAF formation relative to symptom onset.

## 1. Introduction

Stargardt disease (STGD1, OMIM#248200) is one of the most common monogenic inherited retinal diseases [1,2]. The growth rate (GR) of retinal pigment epithelial atrophy, known as definitely decreased autofluorescence (DDAF), is a widely accepted clinical trial endpoint [3]. Variable DDAF GRs have been attributed to their dependence on baseline lesion size [4]. Square root area (SRA) transformation, however, eliminates this dependence and allows linear modelling of GR [5]. A meta-analysis of seven studies showed a bimodal distribution in the effective radius GR peaking at 0.029 and 0.110 mm/year (overall mean of 0.104 mm/year), suggesting two potential subpopulations related to genetic heterogeneity [6]. We recently reported genotype-dependent variation in DDAF GR using ultra-widefield (UWF) AF, where patients harbouring mild or hypomorphic variants showed a significantly slower SRA GR as compared to those with biallelic severe variants or those with one intermediate and one severe variant in trans [3]. Herein we expand on this work by evaluating the effective radius GR in five genotype-specific cohorts to elucidate the basis of this bimodal distribution in GR.

## 2. Materials and Methods

### 2.1. Study Design 

Data were collected retrospectively and prospectively from June 2011 to August 2021. Informed consent was obtained prior to recruitment, and patients were assessed for six months. Sex, age at enrolment and symptom onset were recorded. Patients generally presented within 1-2 years of developing a central scotoma, distortion or paracentral visual loss. Therefore, any error in recall is likely to be less than 2 years. DNA was collected by the Australian Inherited Retinal Disease Registry and DNA Bank and analysed by Molecular Vision Laboratory, Oregon, US [7]. Patients with c.6079C>T, c.[2588G>C;5603A>T], c.3113C>T, c.5882G>A or c.5603A>T (allele 1) in trans with pathogenic or likely pathogenic, intermediate to severe *ABCA4* alleles (allele 2) who had two or more DDAF measurements were included. These 5 variants (allele 1) were assigned mild or intermediate severities based on published clinical data or in vitro assays [8,9,10]. 

### 2.2. Ethical Approval

We adhered to the tenets of the Declaration of Helsinki, and ethics was obtained from the Human Ethics Office of Research Enterprise, the University of Western Australia (RA/4/1/7916, 2021/ET000151).

### 2.3. Imaging Procedures and Outcome Measures

For imaging procedures, first, 30° × 30° field short-wavelength AF images were captured (HRA2, Heidelberg Engineering, Heidelberg, Germany), and the DDAF boundary was outlined (R.C.H.J. and F.K.C.) using Heidelberg Explorer^TM^. DDAF was defined as a region of darkness of at least 90% of that pertaining to the optic disc area. The area was transformed to an effective radius by √(area/π). Serial measurements of the effective radius were plotted against time for linear regression to derive the slope (radius GR) and the x-intercept (age at DDAF formation) for each eye. The times at first or second eye DDAF formation relative to the age at symptom onset (ΔT1 or ΔT2) were calculated. 

### 2.4. Statistical Analysis

Mixed model analysis of variance (ANOVA) assessed differences in radius GR (mm/year) between the left and right eye across 5 genotypes via the eye×genotype interaction. The main effects were investigated if the interaction effect was not significant. Paired sample *t*-tests were performed to determine if differences between symptom onset and age at first or second eye DDAF formation (ΔT1 or ΔT2) were significant. One-way ANOVA determined if there was a difference in ΔT1 and ΔT2 across the 5 genotypes. The least significant difference (LSD) post hoc test assessed pairwise differences. Modelling results were summarised by the mean, standard error (SE) and 95% confidence intervals (CI). Significance was assumed if *p* < 0.05. All analyses were performed with IBM SPSS version 27.

## 3. Results

**Figure 1:**
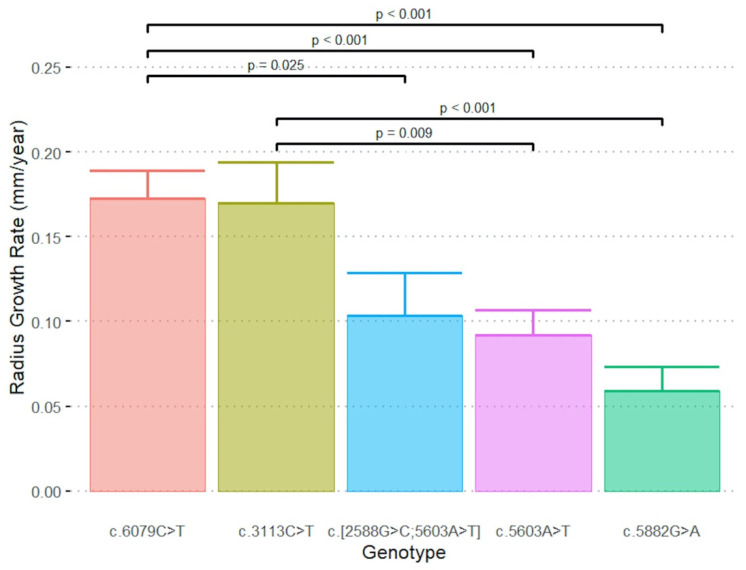
Post hoc results across the five genotype groups in which effective radius growth rates of genotypes c.6079C>T and c.3113C>T were significantly greater than the other groups.

Seventy-five eyes from 39 patients (17 male [44%]; mean±SD age 45 ± 19 years; range 21–86) were followed for a mean ± SD of 4.6 ± 3.1 years. Supplementary Table S1 shows the baseline characteristics, DDAF area, radius and GR by genotype group. A summary of our cohort’s *ABCA4* variants and their respective severities is shown in Supplementary Table S2. No significant eye×genotype interaction (*p* = 0.128) was observed from the mixed ANOVA, nor were there significant differences in radius GR between the right and left eye (*p* = 0.478). Significant differences in the mean radius GR amongst genotypes were noted (*p* < 0.001). The mean radius GR (both eyes) across the five genotypes is shown in Table 1. Figure 1 illustrates post hoc results in which the radius GR of genotype c.6079C>T was significantly greater than all other groups except c.3113C>T. 

**Figure 2:**
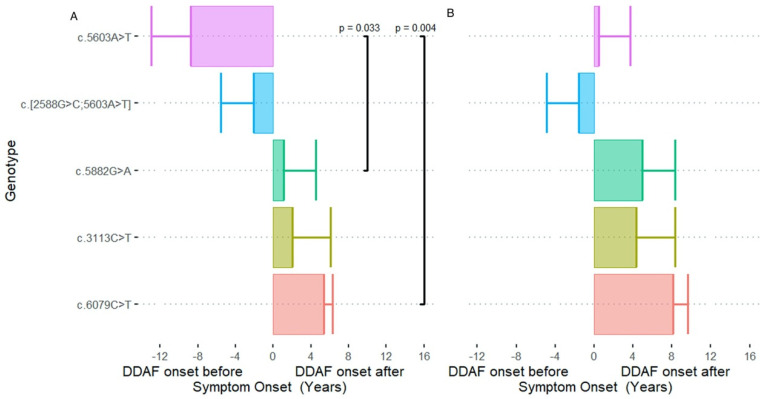
Significant differences in time differences from age at symptom onset to age at (**A**) first or (**B**) second eye DDAF formation (ΔT1 or ΔT2) were observed among the five genotype groups. For c.5603A>T, first eye DDAF formation developed 8.7 years prior to symptom onset, whilst in the second eye, DDAF formation occurred at around the same time as symptom onset. Conversely, the first and second eye DDAF formation developed 5.4 and 8.1 years after onset of symptoms in c.6079C>T.

Overall mean ± SD ΔT1 and ΔT2 were −0.39 ± 10.56 (*p* = 0.821) and 3.97 ± 8.74 (*p* = 0.008) years respectively. Significant differences in ΔT1 were observed among the five genotype groups (*p* = 0.049). For c.5603A>T, first eye DDAF formation developed 8.7 years before, whilst second eye DDAF formation occurred 0.5 years after the onset of symptoms (Figure 2). The reverse trend was generally true for the other genotypes, with c.6079C>T (*p* = 0.004) and c.5882G>A (*p* = 0.033) showing symptom onset preceding DDAF formation by 5.4 and 1.1 years in the first eye, respectively (Table 1). Notably, no significant differences in ΔT2 were noted across the five genotypes (*p* = 0.368).

## 4. Discussion

A meta-analysis by Shen et al. revealed a non-Gaussian distribution of effective radius GR in pooled data from 228 eyes, which they attributed to two Gaussian curves despite three peaks on the histogram [6]. We observed c.6079C>T and c.3113C>T groups to exhibit a similar radius GR of 0.17 mm/year in contrast to three other variants with radius GRs of 0.06–0.10 mm/year. This was based on linear regression with a mean of 4.4 ± 1.5 visits in contrast to only two visits in the meta-analysis. A retrospective cohort of 28 patients by Cicinelli et al. found genotype was not associated with DDAF lesion growth [11]. However, only two genotype groups—(1) one null variant or (2) with two missense variants—were considered, and they did not calculate effective radius GR to control for baseline lesion size. 

Our study sheds light on conflicting functional assessments for c.[2588G>C;5603A>T] and c.3113C>T. Consistent with our results for radius GR, Curtis et al. assigned c.[2588G>C;5603A>T] as a mild complex variant with a relatively high level of expression and basal ATPase activity [9]. Sun et al. demonstrated intermediate and mild impairment of basal ATPase activity in c.2588G>C (simplex) through the two protein products Gly863Ala and Gly863del, respectively [11]. However, they did not assess the combined effect of p.[Gly863Ala, Gly863del; Asn1868Ile]. Despite the severity of this complex variant being assigned as intermediate based on functional [10] (electroretinography) and structural [12] (UWF imaging) assessments, we observed a significantly lower radius GR for c.[2588G>C;5603A>T] as compared to c.6079C>T [3,10]. Therefore, we suggest the severity of the c.[2588G>C;5603A>T] variant is more similar to c.5603A>T than c.6079C>T. Controversy remains regarding the severity of c.3113C>T. Garces et al. reported reduced expression (70% wild type) levels and a lower level of retinal binding activity [13]. Zhang et al. showed c.3113C>T had reduced basal ATPase activity but minimal impact on conformation and stability of *ABCA4* [14]. However, we found an unexpectedly greater radius GR in c.3113C>T, similar to c.6079C>T. The discordance between DDAF GR and variant severities, as determined by molecular assays, UWF imaging and ERG, suggests a differential effect of *ABCA4* variants on RPE and photoreceptor survival. 

Shen et al. applied a horizontal translation factor to the mean baseline and final effective radius to derive age at atrophy onset [6]. They demonstrated age at symptom onset was similar to DDAF onset at a study-level. Their approach was criticised given patients with DDAF may be asymptomatic, whilst those with symptoms may not have evidence of atrophy [15]. Although we showed symptom onset coincided with the predicted age of DDAF formation in the first eye overall, symptom onset was delayed by 8 years in c.5603A>T in contrast to c.6079C>T, where symptom onset occurred 5 years prior to DDAF formation. This is consistent with c.5603A>T manifesting a distinct phenotype of foveal sparing atrophy and c.6079C>T presenting with cone dystrophy. Given age at onset of symptoms is subject to recall bias and is dependent on the extent of foveal involvement, the approach by Shen et al. may be useful particularly in those patients with long-standing disease.

Limitations include a small sample size and retrospective design. Significantly lower numbers of follow-up were obtained for certain genotypes. DDAF area was not adjusted for axial length, and this may have led to minor measurement errors. The effect of the second allele in those carrying c.[2588G>C;5603A>T], c.3113C>T or c.6079C>T may contribute to variability in the age of DDAF formation or its GR. However, most of these subjects (88%) had a second allele that has been previously classified as severe in their effect on *ABCA4* function (Supplementary Table S2). Although the measurement of the DDAF area may serve as one objective endpoint, further studies are required to determine its correlation with other structural and functional measures such as OCT macular volume analysis [16] and microperimetry [17]. Finally, we were unable to obtain visual acuity measurements at the estimated age of first and second eye atrophy onset to examine the relationship between atrophy formation and visual acuity change. 

Genotype plays a significant role in determining the effective radius GR with an almost three-fold difference in lesion growth. DDAF formation preceded symptom onset in those harbouring the hypomorphic *ABCA4* variant c.5603A>T, whilst patients harbouring the c.6079C>T variant did not develop DDAF until years later. Future clinical trial sample size calculation should consider the effect of genotype heterogeneity. A trial inclusion criterion that sets a minimum DDAF size may generate bias in the case-mix of recruited subjects given the impact of genotype on the timing of DDAF formation relative to symptom onset.

## Figures and Tables

**Table 1:** Summary statistics of radius growth rate (mm/year) and time difference between atrophy formation and symptom onset by genotype.

Genotype	N	Radius Growth Rate (mm / Year)	ΔT1 Mean ± SE (95% CI) (Years)	ΔT2 Mean ± SE (Years)
		Mean ± SE (95% CI)	Mean ± SE (95% CI)	Mean ± SE (95% CI)
c.[2588G>C;5603A>T]	4	0.10 ± 0.02 (0.05, 0.15)	−2.1 ± 3.5 ^†^ (−46.7, 42.5)	−1.6 ± 3.8 ^†^ (−49.4, 46.2)
c.3113C>T	4	0.17 ± 0.02 (0.12, 0.22)	2.0 ± 4.1 (−11.0, 15.0)	4.3 ± 4.0 (−8.3, 16.9)
c.5603A>T	11	0.09 ± 0.01 (0.06, 0.12)	−8.7 ± 4.2 ^‡^ (−18.2, 0.6)	0.5 ± 3.2 ^‡^ (−6.9, 7.8)
c.5882G>A	11	0.06 ± 0.01 (0.03, 0.09)	1.1 ± 3.5 ^‡^ (−6.8, 8.9)	4.9 ± 3.4 ^‡^ (−2.8, 12.6)
c.6079C>T	9	0.17 ± 0.02 (0.14, 0.21)	5.4 ± 0.9 (3.3, 7.4)	8.1 ± 1.5 (4.7, 11.6)

SE = Standard Error; CI = confidence Interval. ΔT1 = age at predicted DDAF onset in the first eye—age at symptom onset. ΔT2 = age at predicted DDAF onset in the second eye—age at symptom onset. A negative/positive value for ΔT indicates DDAF formation prior to/after symptom onset. ^†^ Two subjects were excluded due to lack of symptoms and only one eye had DDAF measurements. ^‡^ One subject was excluded due to lack of DDAF measurements in both eyes.

## Supplementary Materials

The following are available online at https://www.mdpi.com/article/10.3390/genes12121981/s1, Table S1: Comparison of atrophy area by genotype group. Table S2: Summary of genetic variants and their severities.
