# Supplementary material for: Genotype-Specific Lesion Growth Rates in Stargardt Disease
Source: Genes (Basel). 2021 Dec 14;12(12):1981. doi: 10.3390/genes12121981 (PMC8701386; doi:10.3390/genes12121981)
Supplement: Supplementary file 1 [file genes-12-01981-s001.zip › genes-1479134-supplementary.pdf]

Supplementary Table S1. Comparison of atrophy area by genotype group

Genotype	Patient No.	Sex	Age at symptom onset (yrs)	Age at examination (yrs)	Number of imaging exams	Follow-up duration (yrs)	Baseline DDAF Area (mm^2)		Baseline DDAF Radius (mm)		DDAF Area growth rate (mm^2/year)		DDAF Radius growth rate (mm/year)	
							Right	Left	Right	Left	Right	Left	Right	Left
5603A>T	1	F	75	75	5	3.0	0.35	5.86	0.33	1.37	0.10	0.80	0.04	0.09
5603A>T	2	M	82	83	3	1.0	11.61	14.45	1.92	2.14	1.80	3.11	0.14	0.22
5603A>T	3	F	32	36	2	2.6	0.08	0.05	0.16	0.13	0.03	0.04	0.02	0.04
5603A>T	4	F	28	40	6	7.6	0.49	0.55	0.39	0.42	0.53	0.26	0.11	0.06
5603A>T	5	F	60	86	2	1.0	29.51	36.4	3.06	3.40	0.95	3.38	0.05	0.15
5603A>T	6	M	81	81	6	5.1	0.96	0.55	2.08	0.81	0.21	0.60	0.05	0.09
5603A>T	7	M	48	58	6	4.0	7.37	1.53	3.78	1.10	0.61	0.47	0.06	0.06
5603A>T	8	M	81	81	3	3.6	2.24	N/A	0.84	N/A	1.10	N/A	0.16	N/A
5603A>T	9	M	46	70	3	6.5	1.15	0.60	13.1	2.04	0.41	2.51	0.10	0.16
5603A>T	10	F	72	69	6	5.0	0.07	0.02	0.15	0.08	0.09	0.08	0.05	0.08
5603A>T	11	M	50	53	6	5.5	0.29	1.00	0.30	0.56	0.06	0.50	0.02	0.10
3113C>T	12	M	62	58	6	4.7	0.85	1.38	0.52	0.66	1.07	0.82	0.18	0.13
3113C>T	13	F	27	47	6	5.3	21.02	20.07	2.59	2.53	2.83	2.90	0.15	0.16
3113C>T	14	M	30	44	6	3.1	1.38	1.42	0.66	0.67	1.58	1.61	0.23	0.24
3113C>T	15	F	17	32	5	2.6	3.91	5.83	1.12	1.36	1.34	1.03	0.16	0.11
5882G>A	16	M	18	24	5	3.1	0.06	0.12	0.14	0.20	0.02	0.04	0.02	0.02
5882G>A	17	M	21	40	5	3.6	3.67	0.98	1.08	0.56	0.54	0.41	0.07	0.09
5882G>A	18	F	15	38	3	1.2	0.08	0.06	0.16	0.14	0.06	0.11	0.05	0.09
5882G>A	19	F	15	36	3	1.2	1.83	2.16	0.76	0.83	0.85	0.32	0.16	0.06
5882G>A	20	F	12	25	2	2.0	0.22	N/A	0.26	N/A	0.13	N/A	0.06	N/A
5882G>A	21	F	25	34	5	2.5	0.14	0.12	0.21	0.20	0.14	0.27	0.07	0.12
5882G>A	22	F	14	27	4	6.0	0.19	0.36	0.25	0.34	0.10	0.11	0.04	0.04
5882G>A	23	M	33	33	2	13.6	0.19	0.16	0.25	0.23	0.04	0.04	0.02	0.02
5882G>A	24	F	31	32	3	10.1	0.07	0.05	0.15	0.13	0.06	0.04	0.03	0.02
5882G>A	25	F	27	43	3	13.9	2.25	2.14	0.85	0.83	0.16	0.24	0.02	0.04
5882G>A	26	F	24	32	4	1.5	0.24	0.19	0.28	0.25	0.20	0.17	0.09	0.09
2588G>C	27	M	19	24	6	7.3	0.15	0.18	0.22	0.24	0.18	0.22	0.06	0.07

2588G>C	28	F	32	28	6	6.0	0.09	0.08	0.17	0.16	0.20	0.17	0.08	0.07
2588G>C	29	F	N/A	43	3	1.5	3.68	4.42	1.08	1.19	1.31	1.52	0.17	0.18
2588G>C	30	F	17	49	4	2.9	18.66	N/A	2.44	N/A	1.37	N/A	0.09	N/A
c.6079C>T	31	M	7	21	3	0.8	1.18	0.97	0.61	0.55	0.83	0.77	0.19	0.19
c.6079C>T	32	F	11	24	4	7.0	3.17	4.09	1.00	1.14	1.70	0.94	0.17	0.11
c.6079C>T	33	F	13	45	5	7.0	25.61	21.92	2.86	2.64	1.89	1.67	0.10	0.09
c.6079C>T	34	M	16	29	7	4.7	8.86	8.02	1.68	1.60	3.05	3.05	0.22	0.23
c.6079C>T	35	M	17	27	3	8.5	5.20	6.05	1.29	1.39	3.66	4.60	0.25	0.28
c.6079C>T	36	F	19	34	4	2.4	3.46	3.51	1.05	1.06	0.74	0.73	0.10	0.10
c.6079C>T	37	M	24	36	5	3.0	0.53	0.78	0.41	0.50	0.57	0.33	0.14	0.08
c.6079C>T	38	F	36	55	3	4.2	11.53	0.61	1.92	0.44	2.00	1.61	0.15	0.26
c.6079C>T	39	M	51	55	7	6.1	0.75	0.14	0.49	0.21	1.57	1.09	0.21	0.22

N/A: not applicable as patient had insufficient data for calculation of radius growth rate.
DDAF, definitely decreased autofluorescence

Supplementary Table S2. Summary of genetic variants and their severities

Patient No.	*ABCA4* Variants					
	Allele 1	Protein	Severity	Allele 2	Protein	Severity
1	c.5603A>T	p.(Asn1868Ile)	mild[1,2]/hypomorphic[1,3]	c.2894A>G	p.(Asn965Ser)	intermediate[1]
2	c.5603A>T	p.(Asn1868Ile)	mild[1,2]/hypomorphic[1,3]	c.2894A>G	p.(Asn965Ser)	severe[1]
3	c.5603A>T	p.(Asn1868Ile)	mild[1,2]/hypomorphic[1,3]	c.[5461-10T>C;5603A>T]	p.[Thr1821Valfs*13, Thr1821Aspfs*6;Asn1868Ile]	severe[6,8]
4	c.5603A>T	p.(Asn1868Ile)	mild[1,2]/hypomorphic[1,3]	c.1643G>A	p.(Trp548*)	severe[9]
5	c.5603A>T	p.(Asn1868Ile)	mild[1,2]/hypomorphic[1,3]	c.1906C>T	p.(Gln636*)	severe[6]
6	c.5603A>T	p.(Asn1868Ile)	mild[1,2]/hypomorphic[1,3]	c.4577C>T	p.(Thr1526Met)	severe[4]
7	c.5603A>T	p.(Asn1868Ile)	mild[1,2]/hypomorphic[1,3]	c.[4670A>G; 6148G>C]	p.[Tyr1557Cys;Val2050Leu]	severe[9]
8	c.5603A>T	p.(Asn1868Ile)	mild[1,2]/hypomorphic[1,3]	c.[2549A>G;4667+5G>T;5882G>A]	p.[Tyr850Cys,?,Gly1961Glu]	severe[9]
9	c.5603A>T	p.(Asn1868Ile)	mild[1,2]/hypomorphic[1,3]	c.4139C>T	p.(Pro1380Leu)	intermediate[4,10]
10	c.5603A>T	p.(Asn1868Ile)	mild[1,2]/hypomorphic[1,3]	c.[4222T>C;4918C>T]	p.[Trp1408Arg;Arg1640Trp]	severe[11]
11	c.5603A>T	p.(Asn1868Ile)	mild[1,2]/hypomorphic[1,3]	c.6031-6044delins18	p.(Ile2003Leufs*41)	severe[9]
12	c.3113C>T	p.(Ala1038Val)	intermediate[2]	c.2564G>A	p.(Trp855*)	severe[9]
13	c.3113C>T	p.(Ala1038Val)	intermediate[2]	c.2564G>A	p.(Trp855*)	severe[9]
14	c.3113C>T	p.(Ala1038Val)	intermediate[2]	c.[3608G>A;4537dup]	p.(Gly1203Glu,Gly1203Aspfs*10;Gln1513Profs* 42)	severe[9]
15	c.3113C>T	p.(Ala1038Val)	intermediate[2]	c.4577C>T	p.(Thr1526Met)	severe[4]
16	c.5882G>A	p.(Gly1961Glu)	mild[2,4,5]	c.4577C>T	p.(Thr1526Met)	severe[4]
17	c.5882G>A	p.(Gly1961Glu)	mild[2,4,5]	c.[4222T>C;4918C>T]	p.[Trp1408Arg;Arg1640Trp]	severe[11]
18	c.5882G>A	p.(Gly1961Glu)	mild[2,4,5]	c.4320del	p.(Phe1440Leufs*6)	severe[9]
19	c.5882G>A	p.(Gly1961Glu)	mild[2,4,5]	c.4320del	p.(Phe1440Leufs*6)	severe[9]
20	c.5882G>A	p.(Gly1961Glu)	mild[2,4,5]	c.5917del	p.(Val1973*)	severe[9]
21	c.5882G>A	p.(Gly1961Glu)	mild[2,4,5]	c.3323G>T	p.(Arg1108Leu)	severe[9]
22	c.5882G>A	p.(Gly1961Glu)	mild[2,4,5]	c.4918C>T	p.(Arg1640Trp)	severe[5,9]
23	c.5882G>A	p.(Gly1961Glu)	mild[2,4,5]	c.4577C>T	p.(Thr1526Met)	severe[4]
24	c.5882G>A	p.(Gly1961Glu)	mild[2,4,5]	c.4577C>T	p.(Thr1526Met)	severe[4]
25	c.5882G>A	p.(Gly1961Glu)	mild[2,4,5]	c.1253T>C	p.(Phe418Ser)	severe[9]
26	c.5882G>A	p.(Gly1961Glu)	mild[2,4,5]	c.[5461-10T>C;5603A>T]	p.[Thr1821Valfs*13, Thr1821Aspfs*6;Asn1868Ile]	severe[6,8]
27	c.[2588G>C;5603A>T]	p.[Gly863Ala,Gly863del; Asn1868Ile]	mild[1]/intermediate[4]	c.[5461-10T>C;5603A>T]	p.[Thr1821Valfs*13, Thr1821Aspfs*6;Asn1868Ile]	severe[6,8]
28	c.[2588G>C;5603A>T]	p.[Gly863Ala,Gly863del; Asn1868Ile]	mild[1]/intermediate[4]	c.3322C>T	p.(Arg1108Cys)	severe[6]
29	c.[2588G>C;5603A>T]	p.[Gly863Ala,Gly863del; Asn1868Ile]	mild[1]/intermediate[4]	c.634C>T	p.(Arg212Cys)	severe[2,6]
30	c.[2588G>C;5603A>T]	p.[Gly863Ala,Gly863del; Asn1868Ile]	mild[1]/intermediate[4]	c.4577C>T	p.(Thr1526Met)	severe[4]
31	c.6079C>T	p.(Leu2027Phe)	intermediate[1,4]	c.4469G>A	p.(Cys1490Tyr)	severe[4]
32	c.6079C>T	p.(Leu2027Phe)	intermediate[1,4]	c.768G>T	p.(Leu257Valfs*17)	severe[6,7]
33	c.6079C>T	p.(Leu2027Phe)	intermediate[1,4]	c.2966T>C	p.(Val989Ala)	intermediate[6]
34	c.6079C>T	p.(Leu2027Phe)	intermediate[1,4]	c.768G>T	p.(Leu257Valfs*17)	severe[6,7]
35	c.6079C>T	p.(Leu2027Phe)	intermediate[1,4]	c.768G>T	p.(Leu257Valfs*17)	severe[6,7]
36	c.6079C>T	p.(Leu2027Phe)	intermediate[1,4]	c.[5461-10T>C;5603A>T]	p.[Thr1821Valfs*13, Thr1821Aspfs*6;Asn1868Ile]	severe[6,8]
37	c.6079C>T	p.(Leu2027Phe)	intermediate[1,4]	c.4139C>T	p.(Pro1380Leu)	intermediate[4,10]
38	c.6079C>T	p.(Leu2027Phe)	intermediate[1,4]	c.4577C>T	p.(Thr1526Met)	severe[4]
39	c.6079C>T	p.(Leu2027Phe)	intermediate[1,4]	c.4577C>T	p.(Thr1526Met)	severe[4]

#c.6031_6044delinsAGTATTTAACCAATATTT

References

1. Curtis, S.B., et al., Functional analysis and classification of homozygous and hypomorphic ABCA4 variants associated with Stargardt macular degeneration. Hum Mutat, 2020. 41(11): p. 1944-1956.
2. Garces, F., et al., Correlating the Expression and Functional Activity of ABCA4 Disease Variants With the Phenotype of Patients With Stargardt Disease. Invest Ophthalmol Vis Sci, 2018. 59(6): p. 2305-2315.
3. Zernant, J., et al., Frequent hypomorphic alleles account for a significant fraction of ABCA4 disease and distinguish it from age-related macular degeneration. J Med Genet, 2017. 54(6): p. 404-412.
4. Fakin, A., et al., The Effect on Retinal Structure and Function of 15 Specific ABCA4 Mutations: A Detailed Examination of 82 Hemizygous Patients. Invest Ophthalmol Vis Sci, 2016. 57(14): p. 5963-5973.
5. Fujinami, K., et al., The clinical effect of homozygous ABCA4 alleles in 18 patients. Ophthalmology, 2013. 120(11): p. 2324-31.
6. Fakin, A., et al., Phenotype and Progression of Retinal Degeneration Associated With Nullizigosity of ABCA4. Invest Ophthalmol Vis Sci, 2016. 57(11): p. 4668-78.
7. Sangermano, R., et al., ABCA4 midigenes reveal the full splice spectrum of all reported noncanonical splice site variants in Stargardt disease. Genome Res, 2018. 28(1): p. 100-110.
8. Tanaka, K., et al., The Rapid-Onset Chorioretinopathy Phenotype of ABCA4 Disease. Ophthalmology, 2018. 125(1): p. 89-99.
9. Heath Jeffery, R.C., et al., Classifying ABCA4 mutation severity using age-dependent ultra-widefield fundus autofluorescence-derived total lesion size. Retina, 2021. [online ahead of print].
10. Garces, F.A., J.F. Scortecci, and R.S. Molday, Functional Characterization of ABCA4 Missense Variants Linked to Stargardt Macular Degeneration. Int J Mol Sci, 2020. 22(1).
11. Shroyer, N.F., et al., Null Missense ABCR (ABCA4) Mutations in a Family with Stargardt Disease and Retinitis Pigmentosa. Investigative Ophthalmology & Visual Science, 2001. 42(12): p. 2757-2761.
